# Vitamin D-Related Risk Factors for Maternal Morbidity and Mortality during Pregnancy: Systematic Review and Meta-Analysis

**DOI:** 10.3390/nu14194124

**Published:** 2022-10-04

**Authors:** María Morales-Suárez-Varela, Nazlı Uçar, José Miguel Soriano, Agustín Llopis-Morales, Beth S. Sanford, William B. Grant

**Affiliations:** 1 Area of Preventive Medicine and Public Health, Department of Preventive Medicine and Public Health, Food Sciences, Toxicology, and Legal Medicine, School of Pharmacy, University de Valencia, 46100 Burjassot, Spain; 2 Biomedical Research Center Network on Epidemiology and Public Health (CIBERESP), Institute of Health Carlos III, 28029 Madrid, Spain; 3 Food & Health Lab, Institute of Materials Science, University of Valencia, 46980 Paterna, Spain; 4 College of Nursing, Rasmussen University, 4012 19th Ave S, Fargo, ND 58103, USA; 5 Sunlight, Nutrition, and Health Research Center, P.O. Box 641603, San Francisco, CA 94164, USA

**Keywords:** maternal mortality, maternal morbidity, preeclampsia, pregnancy, vitamin D deficiency, supplementation, vitamin D, 25-hydroxyvitamin D

## Abstract

Vitamin D deficiency (serum 25-hydroxyvitamin D [25(OH)D] levels <20 ng/mL in serum) is a common health condition among pregnant women, especially in high-risk groups. Evidence has connected vitamin D levels with many health-related problems during pregnancy, including gestational diabetes and preeclampsia. Because of vitamin D’s effect on both mother and fetus, we systematically review the association between 25(OH)D level and its health effects. From a total of 143 studies, 43 came from PubMed, 4 from Cochrane, and 96 from EMBASE. After screening, we identified 38 studies as candidates for inclusion. Ultimately, we limited this review to 23 articles originating from 12 countries, written in English or Spanish, and conducted between 2010 and 2022. We conducted this review according to the Preferred Reporting Items for Systematic Review and Meta-Analysis (PRISMA) guidelines and evaluated the quality and strength of the evidence by using the Navigation Guide Systematic Review Methodology (SING). These systematic reviews summarize findings that support vitamin D’s role in reducing risks of multiple outcomes and the possible contribution of adequate vitamin D levels to a healthy pregnancy.

## 1. Introduction

Vitamin D is a fat-soluble vitamin critical within the body for many functions, including cell proliferation, differentiation, apoptosis, and immune modulation [1]. Vitamin D is transmitted from the mother to the fetus via the placenta and is fundamental at all stages of embryonic and fetal development, from implantation to general growth, including skeletal maturation and placental function [2,3,4].

Worldwide, about 1 million people suffer from vitamin D deficiency (serum 25-hydroxyvitamin D [25(OH)D] < 20 ng/mL, referring to vitamin D_2_ and/or D_3_) [5]. Due to the increased physiological demand for vitamin D during pregnancy, pregnant women are considered a high-risk group for developing vitamin D deficiency (VDD), with prevalence ranging from 51.3% [6] to 100% [7]. VDD in pregnant women increases maternal mortality and morbidity rates. Worldwide, the highest prevalence (>80%) of deficiency in pregnancy was observed among Chinese women (100%) [7] and pregnant Turkish women (95.6%) [8]. In Middle Eastern countries, VDD among pregnant women is an estimated 60–80% [9,10]. Among Iranian pregnant women, studies have reported prevalence rates of 78%, 76%, 70.4%, and 69.2% [11]. The estimated prevalence in pregnant women in the USA and Canada was reported to be 42–72% [12]. In Sweden, a longitudinal study reported 37% of first-trimester pregnant women had 25(OH)D concentrations < 20 ng/mL [13], in comparison with 23% of Canadian women [14]. In Mexico, a previous cross-sectional study reported VDD among 61% of women in the third trimester, and 98% of their newborns had vitamin D deficiency [15].

There are a number of reasons why maternal VDD rates are high during pregnancy. One reason is sun avoidance. In the Middle East, that can be due to wearing concealing clothing as well as not going outdoors during the hot summers. Another reason is that diet provides only a small amount of vitamin D, and then only from animal products, including eggs, fish and meat, unless the food is fortified. Thus, low-latitude countries, which have largely plant-based diets, obtain little vitamin D from food. A third reason is that health care providers do not generally recommend enough vitamin D supplementation in general and during pregnancy in particular.

Vitamin D may impact maternal, fetal, and postnatal growth by affecting calcium absorption [16], parathyroid hormone expression [17], phosphate metabolism [18], growth plate function [19], and possibly regulating the insulin-like growth factor axis [20]. VDD during pregnancy has therefore been associated with adverse health outcomes in the mother, including increased risk of preeclampsia, glucose intolerance, gestational diabetes, preterm birth, and hypocalcemia crisis [21], as well as poor fetal skeletal development [22]. Through this review’s comprehensive meta-analysis, we aim to determine the effect of vitamin D supplementation in preventing maternal mortality and morbidity.

## 2. Materials and Methods

### 2.1. PICO Strategy

We used the PICO strategy (Pregnancy, Intake/level, C/D, mOrtality/mOrbidity) to identify potentially relevant studies. In PICO, the question needs to identify the patient or population problem we intend to study, the planned intervention or treatment, the comparison of one intervention with another (if applicable), and the anticipated outcome. Our PICO framework was “Is there more mortality or morbidity in pregnant women with low levels of vitamin D than in those with adequate levels of vitamin D?”, in which P is pregnant women, I is a low intake/level of vitamin D, C is adequate intake/level of vitamin D, and O is pregnancy mortality and morbidity.

### 2.2. Literature Search 

We searched the PubMed, Cochrane and Embase databases; keywords included “pregnancy,” “gestation,” “vitamin D,” “mortality,” “morbidity,” and “review.” First, we performed a literature search to identify publications eligible for inclusion in PubMed, Cochrane and Embase. Keywords included “pregnancy” OR “gestation“ AND “vitamin D” AND “review” AND “mortality” OR “morbidity.” The search was limited to human subjects and English- and Spanish-language articles published between 2010 and January 2022. We recovered 43 studies from PubMed, 4 from Cochrane, and 96 from Embase, for a total of 143 studies.

Results were screened in a three-stage process based on title, abstract, and full-text review in duplicate by reviewers at each stage. Study selections were compared and discrepancies were resolved by discussion with N.U. and M.M.-S.-V. Duplicates and studies not meeting selection criteria were removed at each round. Search results were uploaded in Mendeley to remove duplicates, and the reference list was entered in Excel for study selection. The initial screening identified 38 candidates, of which 23 articles met inclusion and exclusion criteria.

**Figure 1:**
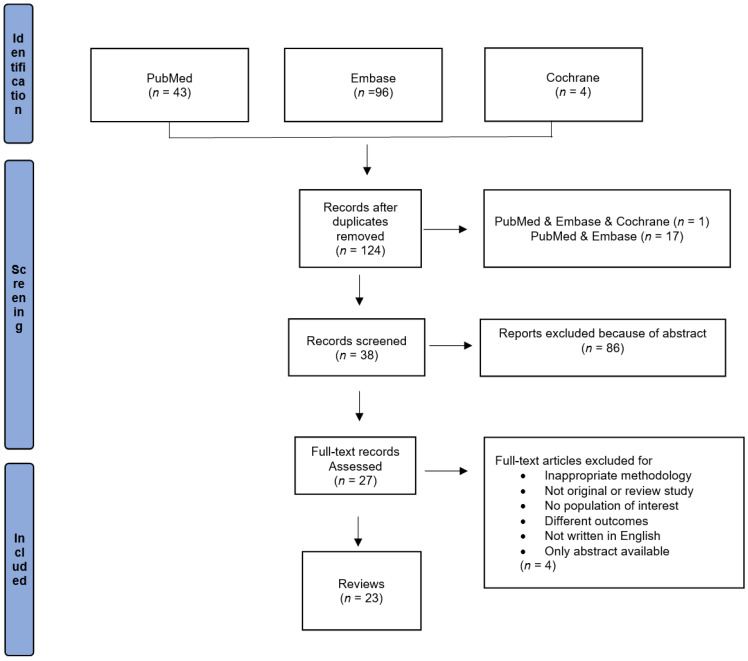
Search and screening strategy for candidate studies.

The Preferred Reporting Items for Systematic reviews and Meta-Analyses (PRISMA) flowchart (Figure 1) shows the number of articles at each stage of the screening process. 

### 2.3. Study Inclusion/Exclusion Criteria and Data Extraction

Studies included in this review met the following criteria: reviews, narrative reviews, clinical review, systematic review, and meta-analysis studies to look at the effects of vitamin D on maternal mortality and morbidity. All studies focused on how vitamin D levels in pregnancy related to maternal mortality and morbidity and were longitudinal in nature. Specific inclusion/exclusion criteria were developed, and only published works meeting all criteria were included. The selection criteria were the following:

1. Review, narrative review, clinical review, systematic review or meta-analysis;
2. Available in English or Spanish;
3. Published between 2010 and January 2022;
4. Study carried out on humans;
5. Exposure of interest is vitamin D status or supplementation during pregnancy;
6. Data on vitamin D or metabolite concentration in maternal blood during pregnancy are available;
7. Main outcomes of interest are the incidence of maternal mortality and morbidity.

After we thoroughly assessed the candidate studies, 23 were included in this meta-analysis. We examined articles to tabulate data, which we summarized under the headings of design, location, vitamin D status, and main findings.

### 2.4. Quality Assessment 

This systematic review and meta-analysis was conducted according to the Preferred Reporting Items for Systematic Reviews and Meta-Analyses (PRISMA) guidelines [23,24]. The systematic review has been registered in the International prospective register of systematic reviews (PROSPERO) (CRD42022343174). 

In addition, to ensure that studies reflected the most current science and evidence-based practice, the Scottish Intercollegiate Guidelines Network (SIGN) was used to ensure rigorous assessment of study quality, validity, bias, and possible confounding variables [25]. Using SIGN ensures a robust assessment of a study’s validity, including key factors such as bias and confounding. SIGN is based on the principles of evidence-based medicine, an approach that ensures using the most up-to-date, reliable, and scientifically solid evidence available in making decisions about a situation being studied [26].

SIGN establishes levels of evidence and recommendations to describe a given study and its results. Levels of evidence are based on study design and the methodological quality of individual studies. Scores are ranked best to worst using 1, 2, 3, and 4, with those scores further ranked with ++, +, and − signs. Grades of recommendation, rated best to worst as A, B, C, and D, are based on the strength of the evidence on which the recommendation is based and do not reflect the recommendation’s clinical importance. 

## 3. Results

### 3.1. Study Characteristics 

Our search yielded 143 studies; 23 review studies remained after further screening. Studies were published between 2010 and 2022. They used data from Chile [27], Canada [28,29], Spain [30,31], Pakistan [32], Brazil [33,34], the United States [35,36,37,38,39,40,41,42], Germany [43], Iran [44,45], India [46], Puerto Rico [47], Poland [48], and Australia [49]. The chosen studies were analyzed according to design, location, vitamin D concentration and supplementation, major findings, and SIGN scores. Table 1 summarizes the study characteristics.

Overall, most included studies were deemed high quality. With the SIGN scores, the 23 review articles could be regarded as good quality. The risk of bias was generally low, with at least 75% of judgments assessed as low risk for four domains: blinding of participants, personnel and outcome assessment, selective reporting, and other biases.

### 3.2. Review and Meta-Analysis Studies

Twenty-three reviews reported analyses for vitamin D supplementation [27,28,29,30,31,32,33,34,35,36,37,38,39,40,41,42,43,44,45,46,47,48,49]. Vitamin D supplementation is is protective for pregnant women, having a significant effect on the incidence of preeclampsia [27,28,30,33,35,44,46] and reduced risk of preterm births [29], prematurity [30], gestational diabetes [31,39,41], and both maternal and infant infections [40].

Through a meta-analysis of observational studies, we suggest that vitamin D supplementation acts as a protective factor for preeclampsia and prematurity [30]. One meta-analysis [30] reviewed cohort studies evaluating the association between vitamin D and prematurity. Four studies used concentrations <75 nmol/L, and six studies considered concentrations <50 nmol/L as vitamin D cutoff points. Results of the subgroup analysis of cohort studies showed a significant association between maternal vitamin D and preterm birth only for concentrations <75 nmol/L: pooled odds ratio (OR) = 1.56; 95% confidence interval (CI), 1.25–1.94; *I*^2^ = 70%; *p* = 0.02. For concentrations <50 nmol/L, pooled OR = 1.09; 95% CI, 0.91–1.30; *I*^2^ = 91%; *p* < 0.00001.

In Oh, Keats, and Bhutta [29], the risk for preterm birth may have been reduced by 36% through vitamin D supplementation (average relative risk (RR) = 0.64; 95% CI, 0.40–1.04; studies = 7), though the upper limit of the confidence interval just crossed the line of no effect. In Tabesh and colleagues [45], a significant association was found between VDD and risk of preeclampsia; however, significant between-study heterogeneity was found (*I*^2^ = 52.7%; *p =* 0.03). In Palacios and colleagues [47], data from original studies involving 446 women showed probable gestational diabetes risk reduction with vitamin D supplementation in comparison with no intervention or placebo groups (RR = 0.51; 95% CI, 0.27–0.97; moderate-certainty evidence). In addition, vitamin D supplementation probably makes little or no difference in risk of gestational hypertension in comparison with no intervention or placebo (*n* = 1130; RR = 0.78; 95% CI, 0.41–1.49).

## 4. Discussion

The most common causes of maternal mortality are severe bleeding, infections/sepsis, embolisms (blockage in the heart or lungs), stroke (can be a blown blood vessel or an embolus), blood pressure disorders (preeclampsia/eclampsia), and gestational diabetes. According to the WHO, the leading causes of maternal mortality (nearly 75%) are severe bleeding, postnatal infections, blood pressure disorders, and complications of labor and delivery [49]. In the US, the top causes of maternal mortality are hemorrhage, cardiovascular complications, infections/sepsis, embolism (pulmonary embolism or other embolisms), cerebrovascular accidents, and noncardiovascular medical conditions such as gestational diabetes mellitus (GDM) [51].

In this review, we aimed to evaluate the current evidence on the effect of vitamin D and vitamin D supplementation on maternal mortality and morbidity. Interventions yielded significant effects, albeit with sparse evidence in some areas.

In recent years, scientific interest has increasingly focused on the consequences of VDD on pregnant women, in particular, the impact of its deficiency on adverse maternal health outcomes. With all significant effects taken together, vitamin D supplementation was associated with a reduced risk of maternal mortality and morbidity-related outcomes.

Many observational studies did report that vitamin D levels were associated with adverse maternal, fetal, and neonatal outcomes, including increased risk of developing preeclampsia, preterm labor, gestational diabetes, being small for gestational age, low birth weight, an increased rate of Cesarean delivery, and infertility [50]. Since vitamin D RCTs have not yielded much useful information regarding the role and requirements for vitamin D for many health outcomes while observational studies have [52], results from observational studies are highlighted in the following paragraphs.

### 4.1. Hemorrhage

Low maternal 25(OH)D concentrations have been found to be associated with an increased risk of postpartum hemorrhage. An observational study from Taiwan involving 600 pregnant women with 25(OH)D concentrations measured in the 36th week of pregnancy found that 25(OH)D below 30 ng/mL was associated with a factor of four-to-five increased risk of postpartum hemorrhage [53].

### 4.2. Gestational Diabetes

In a meta-analysis of 31 observational trials, low vitamin D levels increased the risk of gestational diabetes by 49 (OR = 1.49; 95% CI, 1.18–1.89) [54]. Another meta-analysis of 24 observational studies showed similar results [22]. Observational studies also have shown VDD in pregnancy increases the risk of preeclampsia and that vitamin D supplementation, with or without calcium, may reduce that risk [55].

### 4.3. Pulmonary Embolism or Other Embolism

The incidence of pulmonary embolism, a common cause of maternal mortality, has been found in many studies to be increased in a state of maternal VDD, and the risk decreased with supplementation [27,28,30,33,35,37,42,43,44,45,46]. Other studies concluded pulmonary embolism was not influenced by supplementation [31] or that the connection was unclear [34].

### 4.4. Preterm Birth Risk

Reports have conflicting findings on the role of vitamin D in reducing preterm birth risk. Some studies identified the association of VDD and the inflammatory response with premature rupture of the amniotic membrane and preterm delivery [56,57]. Pooled analysis of four randomized controlled trials in that study showed no significant effect of vitamin D supplementation in preventing preterm birth. Other publications have reported alterations in the cervicovaginal fluid content of vitamin D and vitamin D binding protein as biomarkers of vaginal inflammation and preterm birth risk several weeks before delivery [58]. A review published in 2017 reported that based on 6 vitamin D RCTs, vitamin D supplementation could significantly reduce the risk of preterm birth (pooled RR = 0.57 (95% CI, 0.36–0.91)) and from 18 observational studies that maternal 25(OH)D < 20 ng/mL was associated with a pooled OR = 1.25 (95% CI, 1.13–1.38) [59]. The best observational study on preterm delivery to date was conducted at the Medical University of South Carolina [60]. A total of 1064 consecutive pregnant women were enrolled at their first prenatal visit around the 12th to 14th week of pregnancy. The participants included 488 whites, 395 African Americans, 117 Hispanics, 19 Asians, and 29 multiple or other ethnicities. Their serum 25(OH)D concentration was measured, and they were given bottles of 5000 IU vitamin D3 and counseled on how to achieve 25(OH)D > 40 ng/mL. Achieved 25(OH)D was also measured during pregnancy. Those who achieved >40 ng/mL had a 62% lower risk of PTB compared to those <20 ng/mL (*p* < 0.0001). There was no effect of race/ethnicity on the outcomes.

The journal literature on vitamin D and maternal mortality is relatively limited. However, there is a reasonable body of literature on the role of vitamin D in reducing the risk of adverse pregnancy and birth outcomes for both the developing fetus and mother, e.g., [61]. The review by Wagner et al. [62] outlines important findings regarding complications, including preterm birth, preeclampsia, and gestational diabetes, as well as adverse effects that appear in early childhood, such as asthma and neurological development. This review also points out that vitamin D regulates gene expression through DNA methylation, which has profound effects on fetal development and life after birth. They point out that pregnant women should supplement with 4000–5000 IU/d vitamin D_3_ and achieve 25(OH)D concentrations > 40 ng/mL.

## 5. Strengths and Limitations

An important strength of this review is that it presents an overview of reviews of the effect of vitamin D on the risk of many risk factors for maternal morbidity and mortality during pregnancy. Table 1 can serve as a starting point for those wanting to know the results to date and can help guide future research efforts. The studies included here show significant methodological differences, including mixed ethnicities and genetic reservoirs, countries, times and conditions of vitamin D evaluation, and different brands and qualities of vitamin D supplements among studies. Those factors all contributed to the heterogeneity of the included studies. A limitation is that we may not have been able to access all publications on the relationship between vitamin D and maternal mortality and morbidity during pregnancy because we limited our analysis to studies published in English and Spanish and available through the PubMed, Cochrane, and Embase databases.

## 6. Conclusions

Our meta-analysis showed evidence to support vitamin D supplementation as a cost-effective public health strategy to minimize adverse maternal health outcomes. Whenever possible, supplementation should be based on initial vitamin D serum levels with the intent to obtain and maintain optimal levels of a minimum of 40 ng/mL throughout the pregnancy for maximum impact [61]. In venues where testing is not affordable or convenient, innovative evidence-based technologies such as the *Vitamin D Deficiency Risk Assessment Quiz* (beta) and the Vitamin D*Calculator can aid providers of prenatal care in assessing individual VDD risk and calculating an individualized evidence-based loading and maintenance doses based on target optimal blood levels of 40 ng/mL, respectively (GrassrootsHealth.net, accessed 15 September 2022) In light of the results of the present review, further studies should be conducted. Randomized, controlled, blinded vitamin D supplementation trials must be conducted with pregnant women using standard nutrient physiological design criteria to ensure homogeneity of study design [62], including vitamin D levels (baseline and at time of birth) for all participants, to facilitate future systematic review and meta-analyses, In addition, RCT design may not be ideal for vitamin D outcomes studies because vitamin D intake is difficult to quantify from other sources, as well as lack of compliance, which can lead to unclear study results. Alternatively, they could be observational studies with vitamin D supplementation as done at the Medical University of South Carolina [59]. These studies must include large enough sample sizes to permit evaluating the prevalence of maternal mortality and morbidity.

## Figures and Tables

**Table 1:** Reviews that show vitamin D-related risk factors for maternal morbidity and mortality during pregnancy.

Ref.	Design	Location	Vitamin Concentration and Supplementation	Findings	SIGN	
					LE	GR
González-Wong et al. 2021 [27]	Narrative review	Chile	Supplementation during pregnancy	Vitamin D may lower risk of PE.	3	C
Kinshella et al. 2021 [28]	Review	Canada	Supplementation during pregnancy	Half of reviews reported that vitamin D supplementation had a significant protective effect on PE incidence; little evidence that vitamin D supplementation affected PTB or stillbirth. Pooled outcomes showed 38% reduced risk of developing PE among pregnant women who received vitamin D supplementation in comparison with those who did not, without heterogeneity between studies (RR = 0.62; 95% CI, 0.43–0.91; *I*^2^ = 0%; 12 studies, *n* = 1353), which did not change in direction or significance with sensitivity analyses.	1 ^++^	A
Aguliar-Cordero et al. 2020 [30]	Systematic review and meta-analysis of observational and interventional studies	Spain	Maternal concn during pregnancy	Interventional studies indicated that vitamin D supplementation acts as a prevention factor for PE and prematurity. Observational studies showed that vitamin D insufficiency and deficiency are associated with higher risk of developing PE. However, prematurity and vitamin D were associated only for maternal vitamin D concentrations < 75 nmol/L. Random-effects meta-analysis indicated no significant association between vitamin D, PE, and prematurity in either observational or interventional studies.	2 ^++^	A
Oh, Keats, and Bhutta 2020 [29]	Systematic review and meta-analysis	Canada	Micronutrient and vitamin supplementation during pregnancy	Vitamin D supplementation may have reduced risk of PTB by 36% (average RR = 0.64; 95% CI, 0.40–1.04; studies = 7), though the upper limit of the confidence interval just crossed the line of no effect.	1 ^++^	A
Sibtain et al. 2020 [32]	Systematic review	Pakistan	Status during pregnancy and fetoplacental unit	Studies recommended substantial vitamin D supplementation during pregnancy. High-quality randomized controlled trials (RCTs) required to see optimal level of vitamin D.	2 ^++^	A
De Souza and Pisani 2020 [33]	Narrative review	Brazil	Status and risk of PE	Although studies showing relation between vitamin D and lower risk of PE are limited, maternal status of vitamin D seems to influence risk of developing PE. Therefore, vitamin D supplementation in women may improve pregnancy outcomes.	3	C
Palacios, Kostiuk, and Peña-Rosas 2019 [35]	Review	USA	Supplementation during pregnancy	Supplementation with vitamin D alone during pregnancy probably reduces risk of PE (RR = 0.48; 95% CI, 0.30–0.79; 4 trials, *n* = 499) and gestational diabetes (RR = 0.51; 95% CI, 0.27–0.97; 4 trials, *n* = 446) and probably reduces risk of low birthweight (<2500 g; RR = 0.55, 95% CI, 0.35–0.87; 5 trials, *n* = 697) compared with women who received placebo or no intervention. Vitamin D supplementation may make little or no difference in risk of PTB (<37 weeks) compared with no intervention or placebo (RR = 0.66; 95% CI, 0.34–1.30; 7 trials, *n* = 1640), and vitamin D supplementation may reduce risk of severe postpartum bleeding (RR = 0.68; 95% CI, 0.51–0.91; 1 trial, *n* = 1134).	2 ^++^	A
Pilz et al. 2018 [43]	Review of clinical data	Germany	Status during pregnancy and lactation	Based on available evidence derived from RCTs on vitamin D supplementation in pregnancy, this study reported that vitamin D is safe and improves vitamin D and calcium status, thereby protecting skeletal health. Data from RCTs and meta-analyses of RCTs suggest other beneficial effects but are inconsistent on whether vitamin D supplementation improves clinical neonatal or maternal outcomes such as SGA, fetal/infant growth, infant/neonatal mortality, asthma/wheeze, PE, or GDM.	2 ^++^	A
Akbari et al. 2018 [44]	Systematic review and updated meta-analysis	Iran	Status during pregnancy	Based on the forest plot, lower levels of 25(OH)D were significantly associated with risk of PE (fixed and random *p* < 0.001). Women with vitamin D deficiency (<20 ng/mL) at higher risk of PE. Association can be specific up to 90% at 10.60-ng/mL cutoff.	2 ^++^	A
Nandi, Wadhwani, and Joshi 2017 [46]	Narrative review	India	Vitamin D and LCPUFAs and their role in PE development	Vitamin D [1,25(OH)_2_D_3_] induces CBS gene expression while it can suppress the oxidative stress–induced COX-2 upregulation and thromboxane production. On that basis, it is hypothesized that disturbed vitamin D and LCPUFA metabolism influences regulation of the one-carbon cycle, which will trigger inflammation through oxidative stress in PE. That may lead to altered fetoplacental growth and development of PE.	3	C
Wagner et al. 2017 [36]	Review	USA	Status and supplementation during pregnancy	A growing body of observational studies indicated that maternal hypovitaminosis D (<20 ng/mL or <50 nmol/L) is a significant risk factor for adverse neonatal outcomes, including asthma, multiple sclerosis, and other neurological disorders. Results of RCTs of vitamin D supplementation during pregnancy recently showed decreased complications of pregnancy/birth and GDM.	2 ^++^	A
Agarwal, Kovilam, and Agrawal 2018 [37]	Critical review	USA	PE, PTL, GDM, GH	In pregnancy, vitamin D deficiency is associated with increased incidence of adverse maternal and fetal outcomes, primarily PE, GDM, low birth weight, and PTB. Other outcomes still under study; no definite conclusions drawn yet.	2 ^++^	A
Palacios et al. 2016 [47]	Meta-analysis	Puerto Rico	Oral supplementation (alone or with other vitamins and minerals); maternal levels and risk of developing PE, GDM, PTB, impaired glucose tolerance, Cesarean delivery, GH, and other adverse conditions	Data suggest that pregnant women supplemented with vitamin D had significantly higher vitamin D levels than controls (mean difference, 54.7 nmol/L; 95% CI, 36.6–72.9). Two trials showed lower risk of PE (8.9% vs. 15.5%; average risk ratio, 0.52; 95% CI, 0.25–1.05) and two others showed no difference in risk of GDM with vitamin D supplementation. Additionally, three trials showed that supplementation with vitamin D plus calcium reduced risk of PE (5% vs. 9%; average risk ratio, 0.51; 95% CI, 0.32–0.80).	2 ^++^	A
Pérez-López et al. 2015 [31]	Systematic review and meta-analysis of RCTs	Spain	Circulating levels, PE, GDM, SGA, low birth weight, PTB, birth weight, birth length, Cesarean delivery	Circulating vitamin D levels significantly higher at term than in control group (mean difference, 66.5 nmol/L; 95% CI, 66.2–66.7). Birth weight and birth length were significantly greater in vitamin D group; mean difference, 107.6 g (95% CI, 59.9–155.3) and 0.3 cm (95% CI, 0.10–0.41), respectively. Incidence of PE, GDM, SGA, low birth weight, PTB, and Cesarean delivery not influenced by vitamin D supplementation. Across RCTs, doses and types of vitamin D supplements, gestational age at first administration, and outcomes were diverse.	2 ^++^	A
Weinert and Silveiro 2015 [34]	Critical review	Brazil	Low birth weight, growth restriction, respiratory tract infection, altered glucose homeostasis, increased incidence of GDM, PE, bacterial vaginosis	Current state of evidence is controversial for some other endpoints, and actual benefit of vitamin D supplementation in pregnancy remains unclear.	3	D
Pludowski et al. 2013 [48]	Review of recent evidence	Poland	Status during pregnancy	Various health effects of vitamin D deficiency during pregnancy continue to be reported, notably with increased risk of PE, infection, PTL and PTB, Cesarean delivery, GDM.	2 ^++^	A
Girling and Sykes 2013 [38]	Narrative review	USA	Physiology and current management of thyroid dysfunction and the rarer endocrine disorders in pregnancy and includes current guidance on supplementation	Over recent years, awareness of potential adverse effects of vitamin D deficiency has driven guidance for vitamin D supplementation for pregnant and lactating women.	3	D
Tabesh, Salehi-Abargouei, and Esmaillzadeh 2013 [45]	Systematic review and meta-analysis	Iran	Maternal serum and risk of PE	Overall significant association between vitamin D deficiency and risk of PE; however, significant between-study heterogeneity evident (*I*^2^ = 52.7%; *p* = 0.039). In subgroup analysis, overall effect was significant for studies defining vitamin D deficiency as ≤50 nmol/L (20 ng/mL) but not for those that used <38 nmol/L (15.2 ng/mL). Association was seen for “cohort or nested case–control studies” as well as for “cross-sectional or case–control studies” (2.78; 95% CI, 1.45–5.33; *p* = 0.002). For analysis by study location, associations remained significant only for U.S. studies.	2 ^++^	A
Alzaim and Wood 2013 [39]	Critical review	USA	Status during pregnancy and GDM	Suggested that vitamin D deficiency in pregnant women increases risk for GDM. However, that determination is based largely on only six published observational studies and one short-term intervention study with an active analog form of 1,25(OH)_2_ vitamin D. Effect of treating existing vitamin D deficiency on later development of GDM in pregnant women unknown.	2 ^++^	A
Thorne-Lyman and Fawzi 2012 [40]	Systematic review and meta-analysis	USA	Supplementation during pregnancy for maternal, perinatal, or infant health outcomes	Only low-level evidence relates vitamin D supplementation or intake during pregnancy to perinatal and infant health-related outcomes. Emerging evidence suggesting plausible effects on intrauterine growth restriction, PE, and both maternal and infant infections as important outcomes in need of further research in low-income settings.	2 ^++^	A
Senti et al. 2012 [41]	Systematic review	USA	Status during pregnancy and GDM	Study findings consist solely of level 2 evidence for associating maternal vitamin D deficiency with risk of GDM. Five (83%) studies reported inverse relationship between circulating vitamin D levels and markers of glucose homeostasis associated with GDM or increased risk for GDM associated with reduced maternal levels of vitamin D. In one study, researchers did not identify association between vitamin D and GDM but did identify association between higher vitamin D levels and lower fasting glucose and insulin levels.	3	D
Barrett and McElduff 2010 [50]	Narrative review	Australia	Supplementation during pregnancy	Vitamin D’s role in multiple nonclassical metabolic processes, though shown primarily by association studies in human populations, has a possible physiological basis. Considerable evidence associates low maternal vitamin D levels with worse outcomes for both mother and fetus in pregnancy and for the neonate. Whether association between vitamin D status and a wide range of adverse health outcomes is because vitamin D acts as a marker for some other health parameter such as obesity or occurs because of a direct causal relationship usually remains to be determined. Optimal concentration of vitamin D is unclear or at least controversial. RCTs of vitamin D supplementation with measurement of vitamin D to determine baseline status, level achieved on supplementation with appropriate documentation of possible confounders, and assessment of various health outcomes are required. Trying to achieve a vitamin D concentration of >50 nmol/L seems reasonable in most populations, including pregnant women.	3	B
Mulligan et al. 2010 [42]	Narrative review	USA	Metabolism and implications of deficiency in pregnancy and lactation	Vitamin D deficiency is associated with increased prevalence of PE, a common cause of increased mortality rates in pregnancy. Current recommendations for daily vitamin D intake (200 IU) are inadequate to maintain serum levels of vitamin D in the recommended range during pregnancy and lactation. More studies are needed to determine serum levels and degree of supplementation necessary to optimize maternal and fetal outcomes. However, because vitamin D supplementation is simple and cost-effective with a low likelihood of toxicity, we recommend increased supplementation in all pregnant women to keep serum levels of vitamin D in the reference range for adults (>32 ng/mL).	4	D

Abbreviations: 25(OH)D, 25-hydroxyvitamin D; 95% CI, 95% confidence interval; CBS, cystathionine beta synthase; COX-2, cyclooxygenase 2; GDM, gestational diabetes mellitus; LCPUFA, long-chain polyunsaturated fatty acid; PE, preeclampsia; PTL, preterm labor; PTB, preterm birth; RCT, randomized controlled trial; RR = relative risk; SGA, small for gestational age.

## Data Availability

Not applicable.

## References

[1] Kim I., Kim S.S., Song J.I., Yoon S.H., Park G.Y., Lee Y.-W. (2019). Association between vitamin D level at birth and respiratory morbidities in very-low-birth-weight infants. Korean J. Pediatr..

[2] Eremkina A., Mokrysheva N., Pigarova E., Mirnaya S. (2018). Vitamin D: Effects on pregnancy, maternal, fetal and postnatal outcomes. Ter. Arkhiv..

[3] Baqai S., Siraj A., Imran R. (2020). Association of vitamin-d insufficiency during pregnancy with maternal & perinatal morbidity and mortality. Pak. Armed. Forces Med. J..

[4] Thomas D.J., Khan H.U., Jaidev S.P., Hegde P. (2020). A study on vitamin D levels in preterm and term neonates and their mothers. Int. J. Contemp. Pediatrics.

[5] Sankar J., Lotha W., Ismail J., Anubhuti C., Meena R.S., Sankar M.J. (2016). Vitamin D deficiency and length of pediatric intensive care unit stay: A prospective observational study. Ann. Intensiv. Care.

[6] Gur E.B., Gokduman A., Turan G.A., Tatar S., Hepyilmaz I., Zengin E.B., Eskicioglu F., Guclu S. (2014). Mid-pregnancy vitamin D levels and postpartum depression. Eur. J. Obstet. Gynecol. Reprod. Biol..

[7] Song S.J., Si S., Liu J., Chen X., Zhou L., Jia G., Liu G., Niu Y., Wu J., Zhang W. (2013). Vitamin D status in Chinese pregnant women and their newborns in Beijing and their relationships to birth size. Public Health Nutr..

[8] Ates S., Sevket O., Ozcan P., Ozkal F., Kaya M.O., Dane B. (2016). Vitamin D status in the first-trimester: Effects of Vitamin D deficiency on pregnancy outcomes. Afr. Health Sci..

[9] Behjat Sasan S., Zandvakili F., Soufizadeh N., Baybordi E. (2017). The Effects of Vitamin D Supplement on Prevention of Recurrence of Preeclampsia in Pregnant Women with a History of Preeclampsia. Obstet. Gynecol. Int..

[10] Rostami M., Tehrani F.R., Simbar M., Hosseinpanah F., Majd H.A., Khan F.R. (2017). Rationale and Design of Khuzestan Vitamin D Deficiency Screening Program in Pregnancy: A Stratified Randomized Vitamin D Supplementation Controlled Trial. JMIR Res. Protoc..

[11] Sepandi M., Esmailzadeh S., Hosseini M.S., Hashemi S.R., Abbaszadeh S., Alimohamadi Y., Taghdir M. (2020). Prevalence of Vitamin D Deficiency Among Iranian Pregnant Women. Nutr. Diet. Suppl..

[12] Saraf R., Morton S.M., Camargo C.A., Grant C.C. (2016). Global summary of maternal and newborn vitamin D status–a systematic review. Matern. Child Nutr..

[13] Lundqvist A., Sandström H., Stenlund H., Johansson I., Hultdin J. (2016). Vitamin D Status during Pregnancy: A Longitudinal Study in Swedish Women from Early Pregnancy to Seven Months Postpartum. PLoS ONE.

[14] Perreault M., Moore C.J., Fusch G., Teo K.K., Atkinson S.A. (2019). Factors Associated with Serum 25-Hydroxyvitamin D Concentration in Two Cohorts of Pregnant Women in Southern Ontario, Canada. Nutrients.

[15] Ochoa-Correa E.D.C., Garcia-Hernandez P.A., Villarreal-Perez J.Z., Treviño-Garza C., Villarreal L.E.M.-D., Zapata-Castilleja C.A., De La O-Cavazos M.E. (2017). Vitamin D deficiency in Mexican mothers and their newborns. Gac. Médica México.

[16] Christakos S. (2012). Mechanism of action of 1,25-dihydroxyvitamin D3 on intestinal calcium absorption. Rev. Endocr. Metab. Disord..

[17] Kumar R., Thompson J.R. (2011). The Regulation of Parathyroid Hormone Secretion and Synthesis. J. Am. Soc. Nephrol..

[18] Lederer E. (2014). Regulation of serum phosphate. J. Physiol..

[19] Eisman A.J., Bouillon R. (2014). Vitamin D: Direct effects of vitamin D metabolites on bone: Lessons from genetically modified mice. BoneKEy Rep..

[20] Ciresi A., Giordano C. (2017). Vitamin D across growth hormone (GH) disorders: From GH deficiency to GH excess. Growth Horm. IGF Res..

[21] Larqué E., Morales E., Leis R., Blanco-Carnero J.E. (2018). Maternal and Foetal Health Implications of Vitamin D Status during Pregnancy. Ann. Nutr. Metab..

[22] Wei S.-Q., Qi H.-P., Luo Z.-C., Fraser W.D. (2013). Maternal vitamin D status and adverse pregnancy outcomes: A systematic review and meta-analysis. J. Matern. Fetal Neonatal Med..

[23] Moher D., Shamseer L., Clarke M., Ghersi D., Liberati A., Petticrew M., Shekelle P., Stewart L.A. (2015). Preferred reporting items for systematic review and meta-analysis protocols (prisma-p) 2015 statement. Syst. Rev..

[24] Moher D., Liberati A., Tetzlaff J., Altman D.G., PRISMA Group (2009). Preferred Reporting Items for Systematic Reviews and Meta-Analyses: The PRISMA Statement. Ann. Intern. Med..

[25] (2015). SIGN 50: A Guideline Developer’s Handbook.

[26] Sackett D.L. (1997). Evidence-based medicine. Semin. Perinatol..

[27] González-Wong C., Fuentes-Barría H., Aguilera-Eguía R., Urbano-Cerda S., Vera-Aguirre V. (2021). The role of vitamin D in preeclampsia risk: A narrative review. Rev. Chil. Nutr..

[28] Kinshella M.-L., Omar S., Scherbinsky K., Vidler M., Magee L., von Dadelszen P., Moore S., Elango R., The PRECISE Conceptual Framework Working Group (2021). Effects of Maternal Nutritional Supplements and Dietary Interventions on Placental Complications: An Umbrella Review, Meta-Analysis and Evidence Map. Nutrients.

[29] Oh C., Keats E.C., Bhutta Z.A. (2020). Vitamin and Mineral Supplementation During Pregnancy on Maternal, Birth, Child Health and Development Outcomes in Low- and Middle-Income Countries: A Systematic Review and Meta-Analysis. Nutrients.

[30] Aguilar-Cordero M., Lasserrot-Cuadrado A., Mur-Villar N., León-Ríos X., Rivero-Blanco T., Pérez-Castillo I. (2020). Vitamin D, preeclampsia and prematurity: A systematic review and meta-analysis of observational and interventional studies. Midwifery.

[31] Pérez-López F.R., Pasupuleti V., Mezones-Holguin E., Benites-Zapata V.A., Thota P., Deshpande A., Hernandez A.V. (2015). Effect of vitamin D supplementation during pregnancy on maternal and neonatal outcomes: A systematic review and meta-analysis of randomized controlled trials. Fertil. Steril..

[32] Sibtain S., Sinha P., Manoharan M., Azeez A. (2020). Controversies related to vitamin D deficiency effect on the maternal and feto-placental unit–an update. J. Obstet. Gynaecol..

[33] De Souza E.A., Pisani L.P. (2020). The relationship among vitamin D, TLR4 pathway and preeclampsia. Mol. Biol. Rep..

[34] Weinert L.S., Silveiro S.P. (2015). Maternal–Fetal Impact of Vitamin D Deficiency: A Critical Review. Matern. Child Health J..

[35] Palacios C., Kostiuk L.K., Peña-Rosas J.P. (2019). Vitamin D supplementation for women during pregnancy. Cochrane Database Syst. Rev..

[36] Wagner C.L., Hollis B.W., Kotsa K., Fakhoury H., Karras S.N. (2017). Vitamin D administration during pregnancy as prevention for pregnancy, neonatal and postnatal complications. Rev. Endocr. Metab. Disord..

[37] Agarwal S., Kovilam O., Agrawal D.K. (2018). Vitamin D and its impact on maternal-fetal outcomes in pregnancy: A critical review. Crit. Rev. Food Sci. Nutr..

[38] Girling J., Sykes L. (2013). Thyroid disorders and other endocrinological disorders in pregnancy. Obstet. Gynaecol. Reprod. Med..

[39] Alzaim M., Wood R.J. (2013). Vitamin D and gestational diabetes mellitus. Nutr. Rev..

[40] Thorne-Lyman A., Fawzi W.W. (2012). Vitamin D During Pregnancy and Maternal, Neonatal and Infant Health Outcomes: A Systematic Review and Meta-analysis. Paediatr. Périnat. Epidemiol..

[41] Senti J., Thiele D.K., Anderson C.M. (2012). Maternal Vitamin D Status as a Critical Determinant in Gestational Diabetes. J. Obstet. Gynecol. Neonatal Nurs..

[42] Mulligan M.L., Felton S.K., Riek A.E., Bernal-Mizrachi C. (2010). Implications of vitamin D deficiency in pregnancy and lactation. Am. J. Obstet. Gynecol..

[43] Pilz S., Zittermann A., Obeid R., Hahn A., Pludowski P., Trummer C., Lerchbaum E., Pérez-López F.R., Karras S.N., März W. (2018). The Role of Vitamin D in Fertility and during Pregnancy and Lactation: A Review of Clinical Data. Int. J. Environ. Res. Public Health.

[44] Akbari S., Khodadadi B., Ahmadi S.A.Y., Abbaszadeh S., Shahsavar F. (2018). Association of vitamin D level and vitamin D deficiency with risk of preeclampsia: A systematic review and updated meta-analysis. Taiwan. J. Obstet. Gynecol..

[45] Tabesh M., Salehi-Abargouei A., Tabesh M., Esmaillzadeh A. (2013). Maternal Vitamin D Status and Risk of Pre-Eclampsia: A Systematic Review and Meta-Analysis. J. Clin. Endocrinol. Metab..

[46] Nandi A.A., Wadhwani N.S., Joshi S.R. (2017). Altered metabolic homeostasis between vitamin D and long chain polyunsaturated fatty acids in preeclampsia. Med. Hypotheses.

[47] Palacios C., De-Regil L.M., Lombardo L.K., Peña-Rosas J.P. (2016). Vitamin D supplementation during pregnancy: Updated meta-analysis on maternal outcomes. J. Steroid Biochem. Mol. Biol..

[48] Pludowski P., Holick M.F., Pilz S., Wagner C.L., Hollis B.W., Grant W.B., Shoenfeld Y., Lerchbaum E., Llewellyn D.J., Kienreich K. (2013). Vitamin D effects on musculoskeletal health, immunity, autoimmunity, cardiovascular disease, cancer, fertility, pregnancy, dementia and mortality—A review of recent evidence. Autoimmun. Rev..

[49] Barrett H., McElduff A. (2010). Vitamin D and pregnancy: An old problem revisited. Best Pract. Res. Clin. Endocrinol. Metab..

[50] CDC Pregnancy Mortality Surveillance System. 22 June 2022. https://www.cdc.gov/reproductivehealth/maternalinfanthealth/pregnancy-mortality-surveillance-system.htm.

[51] WHO Maternal Mortality. 19 September 2019. https://www.who.int/en/news-room/fact-sheets/detail/maternal-mortality.

[52] Grant W.B., Boucher B.J., Al Anouti F., Pilz S. (2022). Comparing the Evidence from Observational Studies and Randomized Controlled Trials for Nonskeletal Health Effects of Vitamin D. Nutrients.

[53] Li W.-J., Chen K.-H., Huang L.-W., Tsai Y.-L., Seow K.-M. (2022). Low Maternal Serum 25-Hydroxyvitamin D Concentration Is Associated with Postpartum Hemorrhage: A Retrospective Observational Study. Front. Endocrinol..

[54] Aghajafari F., Nagulesapillai T., Ronksley P.E., Tough S.C., O’Beirne M., Rabi D.M. (2013). Association between maternal serum 25-hydroxyvitamin D level and pregnancy and neonatal outcomes: Systematic review and meta-analysis of observational studies. BMJ.

[55] Baker A.M., Haeri S., Camargo A.C., Stuebe A.M., Boggess A.K. (2011). A Nested Case-Control Study of First-Trimester Maternal Vitamin D Status and Risk for Spontaneous Preterm Birth. Am. J. Perinatol..

[56] Bodnar L.M., Rouse D.J., Momirova V., Peaceman A., Sciscione A., Spong C.Y., Varner M., Malone F.D., Iams J.D., Mercer B.M. (2013). Maternal 25-Hydroxyvitamin D and Preterm Birth in Twin Gestations. Obstet. Gynecol..

[57] Thota C., Menon R., Fortunato S.J., Brou L., Lee J.-E., Al-Hendy A. (2014). 1,25-Dihydroxyvitamin D Deficiency Is Associated with Preterm Birth in African American and Caucasian Women. Reprod. Sci..

[58] Liong S., Di Quinzio M.K.W., Fleming G., Permezel M., Georgiou H.M. (2013). Is Vitamin D Binding Protein a Novel Predictor of Labour?. PLoS ONE.

[59] Zhou S.-S., Tao Y.-H., Huang K., Zhu B.-B., Tao F.-B. (2017). Vitamin D and risk of preterm birth: Up-to-date meta-analysis of randomized controlled trials and observational studies. J. Obstet. Gynaecol. Res..

[60] McDonnell S.L., Baggerly K.A., Baggerly C.A., Aliano J.L., French C.B., Baggerly L.L., Ebeling M.D., Rittenberg C.S., Goodier C.G., Niño J.F.M. (2017). Maternal 25(OH)D concentrations ≥40 ng/mL associated with 60% lower preterm birth risk among general obstetrical patients at an urban medical center. PLoS ONE.

[61] Morales-Suárez-Varela M.M., Uçar N., Peraita-Costa I., Huertas M.F., Soriano J.M., Llopis-Morales A., Grant W.B. (2022). Vitamin D-Related Risk Factors for Maternal Morbidity during Pregnancy: A Systematic Review. Nutrients.

[62] Heaney R.P. (2014). Guidelines for optimizing design and analysis of clinical studies of nutrient effects. Nutr. Rev..

